# The Impact of Depression on Defense Mechanisms in Adults: The Moderating Role of Attachment Style

**DOI:** 10.3390/bs16010057

**Published:** 2025-12-29

**Authors:** Andra-Iuliana Tanase, Amelia-Damiana Trifu, Simona Trifu

**Affiliations:** 1 Faculty of Psychology and Educational Sciences, University of Bucharest, 050663 Bucharest, Romania; andra-iuliana.tanase68@s.fpse.unibuc.ro; 2 Department of General Medicine, “Carol Davila” University of Medicine and Pharmacy, 020021 Bucharest, Romania; 3 Department of Clinical Neurosciences, “Carol Davila” University of Medicine and Pharmacy, 020021 Bucharest, Romania; simona.trifu@umfcd.ro

**Keywords:** depression and defense mechanisms, attachment styles and depression, defensive functioning in mental health, gender differences in defense mechanisms, psychodynamic factors in depression

## Abstract

Depressive disorders are strongly influenced by personality organization, attachment style, and defensive functioning. This study examined the associations between depression severity, defense mechanisms, and adult attachment styles, and explored potential moderating effects of gender. A community sample completed standardized measures assessing depressive symptoms, defense mechanisms (mature, neurotic, immature), and attachment dimensions (anxious, avoidant). Correlational and regression analyses indicated that higher depressive severity was negatively associated with denial and dissociation, while no significant links emerged for projection or mature defenses. Anxious attachment predicted greater use of projection (B = 4.65, *p* = 0.040), but depression did not moderate this association. Cluster analysis identified two distinct profiles: one with moderate depression and higher denial, and another with severe depression and markedly lower denial. Men reported higher dysfunctional defenses overall, whereas a significant depression × gender interaction suggested that depressive severity was associated with reduced dysfunctional defenses among women (B = −0.58, *p* = 0.002). These findings challenge prevailing evidence that depressive severity correlates with greater immature defense use, instead suggesting a possible defensive collapse at high symptom levels. The study contributes novel insights into how attachment and gender shape defensive functioning in depression, emphasizing the need for longitudinal and clinical replication.

## 1. Introduction

Depressive disorders remain a major global public health challenge, with pervasive impacts on emotional functioning, interpersonal relationships, and overall adaptive capacities. Contemporary research increasingly emphasizes not only symptom severity, but also underlying processes of emotion regulation, interpersonal style, and unconscious coping strategies, as these may shape onset, maintenance, and recovery from depression. Two psychological constructs in particular-defense mechanisms and attachment styles-have emerged as promising links between intrapsychic and relational functioning on the one hand, and depressive symptomatology on the other. Yet despite growing interest, the interplay among depressive severity, defensive functioning, and attachment style remains incompletely understood.

Defense mechanisms-defined historically as relatively automatic, unconscious psychological operations that mediate internal conflicts and external stressors—have a long tradition in psychodynamic theory (Bailey & Pico, 2023; Freud & Institute of Psycho-analysis (Great Britain), 1993). They are commonly organized in hierarchical models of adaptiveness (e.g., mature, neurotic, immature/disavowal) and have been linked to a wide range of clinical phenomena, including depression. Several recent meta-analyses and reviews confirm that individuals with depressive disorders tend to employ more immature defenses and fewer mature defenses compared to non-depressed peers. For example, a 2024 systematic review and meta-analysis of defensive functioning in depression found that lower overall defensive functioning and higher reliance on maladaptive defensive styles were robustly associated with depressive severity (Fiorentino et al., 2024). This meta-analysis also showed that defensive functioning tends to improve over the course of psychological treatment, underscoring its clinical relevance as a modifiable process rather than a fixed trait. Complementing this, network-analysis work in non-clinical samples has shown that defense mechanisms themselves form complex interconnections, and that certain immature defenses (e.g., passive aggression, denial, projection) may occupy central positions in networks of depressive and anxious symptoms (Di Giuseppe et al., 2024). This suggests that specific defenses may play a disproportionately influential role in maintaining emotional distress. Beyond meta-analytic evidence, several recent studies have replicated the association between immature defensive functioning and depressive severity across diverse populations. Network analyses in bereaved adults and individuals with mixed depressive and anxiety symptoms showed that immature defenses, such as passive aggression and acting out, are tightly interconnected with depression and anxiety scores (Di Giuseppe et al., 2024; Giunta et al., 2025). Large national data also indicate that major depressive and bipolar disorders are characterized by greater reliance on immature defenses than anxiety disorders, and that higher immature defense use is associated with poorer psychosocial functioning (Blanco et al., 2025). In myocardial infarction patients, heightened depressive and anxiety symptoms similarly co-occur with lower overall defensive functioning and greater use of immature defenses (Cruciani et al., 2025). Longitudinal work further shows that immature defenses decrease in parallel with symptom improvement during psychotherapy for depressive and anxiety disorders (Babl et al., 2019; Fiorentino et al., 2024; Scaini et al., 2022).

Attachment theory, originated by Bowlby and Ainsworth, offers an interpersonal–developmental lens: internal working models of self and others, shaped by early caregiver relationships, guide expectations, affect regulation and relational patterns across the lifespan. In adult research, insecure attachment styles (anxious/preoccupied or avoidant/dismissing) have been reliably associated with heightened vulnerability to depression, impaired interpersonal functioning, and altered emotion regulation. A recent large meta-analysis including over 79,000 participants confirmed that both attachment anxiety and attachment avoidance are moderately to strongly associated with depressive symptoms, with attachment anxiety showing the stronger association (Zhang et al., 2022). These associations remain robust across age groups, cultures, and clinical severity levels. For instance, anxious attachment is characterized by hyperactivation of the attachment system, fears of abandonment or rejection, and a proclivity toward seeking closeness, whereas avoidant attachment tends toward suppression of attachment needs and emotional distancing (Zuckerman et al., 2023). The relation between attachment and defense mechanisms has also received increasing attention: recent studies indicate that individuals with anxious attachment tend to use more neurotic and immature defenses, while avoidant attachment similarly correlates with less adaptive defensive functioning. Theoretically, attachment representations reflect relational templates for managing safety and distress, whereas defense mechanisms constitute intrapsychic strategies for regulating emotional conflict—together forming a unified system of implicit emotion regulation. Empirically, several recent studies support this integration. For example, Bayram Şahin et al. (2024) found that immature defenses significantly predicted both anxious (r ≈ 0.44) and avoidant (r ≈ 0.39) attachment, whereas secure attachment correlated negatively with immature defenses (r ≈ −0.29) (Bayram Şahin et al., 2024). Similarly, Ciocca et al. (2020) showed that the relationship between insecure attachment and psychological distress is partially mediated by immature defenses, indicating that defensive functioning may serve as a key mechanism linking attachment insecurity to depressive symptomatology (Ciocca et al., 2020).

Despite these parallel studies, key gaps remain unresolved. First, much of the research treats defensive functioning and attachment style in isolation (Cruciani et al., 2025; Di Giuseppe et al., 2024; Ma et al., 2024) rather than examining their joint contributions to depression: how do attachment and defense mechanisms interact in the context of depressive symptoms? Second, while many studies report positive linear associations between depressive symptoms and immature defenses (i.e., more depression → more immature defenses) (Fiorentino et al., 2024; Ma et al., 2024; Scaini et al., 2022), fewer have explored potential non-linearities, threshold effects or how defensive style may shift across levels of severity (Høglend & Perry, 1998; Mrozowicz-Wrońska, 2023; Perry & Bond, 2012). Third, although gender differences in both attachment patterns and defense mechanisms are suggested-such as women’s greater tendency toward internalizing defenses and men’s greater use of externalizing defenses-the moderating role of gender in the depression–defense–attachment nexus is seldom examined in depth. Most previous research relies on variable-centered approaches (correlations, regressions), whereas person-centered approaches, such as cluster analysis, which may better capture the heterogeneity of depressive profiles, are still rare.

In response to these conceptual and empirical gaps, the present study sought to: explore the associations among depressive symptom severity, specific defense mechanisms (e.g., denial, dissociation, projection), and adult attachment styles (anxious, avoidant); examine whether depressive severity moderates the link between attachment style and particular defenses; use data-driven clustering to identify profiles of participants across combinations of depression severity, attachment style, and defense usage; and assess gender differences and the interaction of gender with depression and defensive functioning. By integrating psychodynamic and attachment-based perspectives within a single empirical framework, this study seeks to advance a more nuanced understanding of unconscious emotion regulation in depression and to inform more personalized psychotherapeutic interventions.

## 2. Materials and Methods

The research aimed to investigate the relationship between depression and defense mechanisms, identifying the predominant types of mechanisms used by people with depression (H1); examining the role of attachment style as a moderator in the relationship between depression and defense mechanisms (H2); analyzing gender differences in the use of defense mechanisms and their impact on depression (H3); presenting clinical implications for psychotherapeutic interventions based on the identification of attachment styles and the use of defense mechanisms (H4). The sample size (N = 200) was established taking into account the recommendations of the specialized literature for correlational and regression-type statistical analyses, which indicate a minimum of 100–150 participants to ensure adequate statistical power and statistically strong results. The selected sample size (N = 200) is consistent with methodological and empirical recommendations for correlational and regression-based psychological research. Prior work indicates that samples of approximately 100–150 participants are sufficient to detect small-to-moderate effects with acceptable statistical power (Bujang, 2024; Cramer, 2006; Sharma & Sinha, 2010), while samples of around 200 participants provide robust power for interaction effects and multivariate models (Scott et al., 2009; Vaillant, 1994).

The study involved a sample of 200 adults, selected through a non-probability sampling method based on the participants’ willingness to engage in the present study. Participants were recruited from diverse socio-economic backgrounds in terms of education level, aged between 18 and 55 years. Inclusion criteria were age between 18 and 55 years, written informed consent, and willingness to voluntarily participate in the study. Exclusion criteria based on psychiatric history were applied to reflect the diversity of the general population and to observe natural variations in depressive symptoms, defense mechanisms, and attachment styles in the absence of a formal psychiatric diagnosis. 

To measure the variables included in this study, three internationally validated psychometric instruments were used, each having high internal consistency and being frequently used in research: Beck Depression Inventory-II (BDI-II), Defense Style Questionnaire (DSQ-60), Experiences in Close Relationships-Revised (ECR-R). Beck Depression Inventory-II (BDI-II) measures the level of depressive symptoms through 21 items that assess different aspects of depression (affective symptoms, cognitive symptoms, somatic and vegetative symptoms), each item rated on a scale from 0 to 3 (A. T. Beck et al., 1996). Defense Style Questionnaire (DSQ-60), a self-report measure with 60 items, used for the assessment of psychological defense mechanisms, that address each of the 30 individual defense mechanisms of the DSM IV (Thygesen et al., 2008). Experience in Close Relationships-Revised (ECR-R) assesses attachment styles according to the dimensions: anxious and avoidant and has an α = 0.91. The questionnaire consisted of 36 items, each rated on a Likert scale from 1 (strongly disagree) to 7 (strongly agree) (Fraley et al., 2000).

In the present study, internal consistency coefficients (Cronbach’s alpha) were not computed directly from the dataset because only total scale and subscale scores were available for analysis, while the original item-level responses were not retained in the database. Since the estimation of Cronbach’s alpha requires access to individual item variances and inter-item covariances, it was not mathematically possible to calculate internal consistency directly from the present dataset. However, all instruments used in this study have been extensively validated internationally and show consistently strong psychometric properties across clinical and non-clinical samples. For this reason, reliability estimates reported in prior validation research were used as reference indicators of measurement quality.

The Beck Depression Inventory–II (BDI-II) demonstrates excellent internal consistency across diverse populations, with Cronbach’s alpha values typically ranging between α = 0.89 and α = 0.93 in community samples and between α = 0.90 and α = 0.94 in clinical populations. These values indicate a high level of internal homogeneity among the 21 items assessing depressive symptomatology. According to researchers, for Romanian population BDI-II internal consistency α = 0.90 (Mocan et al., 2018).

The Defense Style Questionnaire (DSQ-60) also shows satisfactory to good internal consistency across its major dimensions. Previous studies report Cronbach’s alpha values ranging from approximately α = 0.70 to α = 0.86 for mature, neurotic, and immature defense styles, supporting the reliability of both dimensional and total defense scores. Although reliability tends to vary somewhat across specific defense subscales, the overall structure of the DSQ demonstrates stable psychometric performance in both psychiatric and non-clinical samples. Crașovan and Maricuțoiu (2012) (Romanian validation sample N = 1011): reports Cronbach’s alpha for the 3 higher-order DSQ-60 factors as α = 0.73 (Defensive Style), α = 0.60 (Adaptive Style), α = 0.70 (Affective Adjustment Style) (Crașovan & Maricuțoiu, 2012).

The Experiences in Close Relationships–Revised (ECR-R) questionnaire exhibits excellent reliability for both attachment dimensions. Extensive validation studies consistently report Cronbach’s alpha coefficients above α = 0.90 for attachment anxiety and α = 0.88–0.94 for attachment avoidance, indicating very high internal consistency of the 36-item measure across different languages and cultural contexts. Romanian ECR-R (31-item) reliability coefficients are attachment anxiety: α = 0.91 and attachment avoidance: α = 0.90 (Rotaru & Rusu, 2013). 

Taken together, the strong and stable psychometric properties of the BDI-II, DSQ, and ECR-R reported in the international literature provide confidence in the reliability of the constructs assessed in the present study, despite the absence of item-level reliability estimation within the current sample. This approach is consistent with prior correlational and secondary-data studies in which only total scores were available for analysis.

The data collection procedure was carried out using self-administered questionnaires, which were completed hybrid, either online or on paper. Participants were informed about the purpose of the study and signed informed consent, thus ensuring the confidentiality and anonymity of the data. The collection process lasted around 30–40 min/participant. After collecting the data, they were entered and analyzed in SPSS, using appropriate statistical techniques to test the developed hypotheses. Regarding statistical analysis, the data were analyzed using SPSS software v 24, applying appropriate statistical methods specific to each hypothesis. To evaluate the relationships between variables, Pearson correlation analysis for normal data distribution and multiple regressions were used. Also, the *t*-test for independent samples was performed, where appropriate, to compare the means of the participants in the study. The statistical significance level was set at *p* < 0.05. For example, to test whether the level of depressive symptoms is associated with the type of defense mechanisms used, in the sense that people with higher levels of depression may resort more frequently to maladaptive mechanisms (e.g., denial, projection, dissociation), which supports an association between the level of depressive symptoms and the use of defense mechanisms, Pearson correlation was used. In the case of analysis of the anxious attachment style if it is associated with an increased use of the defense mechanism of projection, which focuses on the analysis of the manifestation of anxious attachment style with projection, moderated regression was applied to verify whether anxious attachment influences the relationship between depression and projection. The variables were associated in this sense to outline the effects of collinearity and facilitate the interpretation of significant interactions.

To test if the formation of clusters in which avoidant attachment and high depression are associated with frequent use of denial, which focuses on the formation of clusters with high levels of depression, avoidant attachment, and frequent use of denial, we used a cluster analysis (k-means). This helps us identify natural clustering patterns in the dataset. Regarding to the fact that women will have higher scores in the use of dysfunctional defense mechanisms compared to men, and depression will amplify this use more in women, which examines gender differences in the use of dysfunctional defense mechanisms and the moderating role of depression on these differences, we applied t-tests for independent samples. These tests compare scores between women and men, and to explore the interaction between gender and the level of depression in the use of these mechanisms, we used ANOVA. The choice of these methods reflects the nature of the variables (nominal, continuous) and the need to test complex relationships between the psychological variables involved in the proposed model.

Given the complexity of the identified relationships and the use of statistical tests, which include interactions in the literature, we considered it essential to check the power of the statistical test to ensure that the conclusions are valid. The power of the test, defined as the probability of rejecting the null hypothesis when it is false, becomes a key element in studies that aim to identify interaction effects between test variables. In this context, we decided to estimate the sample size using the G*Power 3.1 software, which helps us to analytically plan the necessary statistical resources. The parameters for calculation were: expected effect size–estimated based on observed coefficients and literature (100–150) (Cramer, 2006; Sharma & Sinha, 2010; Vaillant, 1994); alpha levels: −α = 0.05, two-tailed; number of predictors–Hypothesis 1 (H1): 1 predictor per correlation (depression); Hypothesis 2 (H2): 3 predictors (anxious attachment, depression, interaction); Hypothesis 4 (H4): 2 predictors (gender, gender × depression interaction); sample size justification: N = 200 considered adequate for detecting small–moderate effects, ensuring a minimum acceptable statistical power (≥0.80). The results showed that the sample size collected is strong to ensure a minimum acceptable statistical power (≥0.80). This methodological approach strengthens the robustness of the interpretation and its quality.

## 3. Results

To assess the impact of depression on defense mechanisms and the moderating role of attachment style, several statistical methods specific to each hypothesis were used. These results contribute to the understanding of how depression, as a psychological disorder, influences the use of defense mechanisms and the way in which attachment styles can influence this effect. Analyzing the interaction between the level of depressive symptoms and the defense mechanisms used by individuals, we expect that people with higher levels of depression will resort more frequently to maladaptive defense mechanisms, such as denial, projection or dissociation. Table 1 illustrates the correlations between the Beck Depression Inventory (BDI-II) and the defense mechanisms measured within the sample selected in the research. The analysis assumed the identification of the compatibility between the level of depressive symptomatology and the use of adaptive (humor, anticipation and sublimation) and maladaptive (projection, denial and dissociation) defense mechanisms. Pearson correlation coefficients were used to provide conclusive information about the direction and strength of the relationship between the two variables tested, and the statistical significance values indicate whether what we assumed is validated. Thus, the table highlights the extent to which certain defense mechanisms are associated with the severity of depression among the participants included in the study.

As shown in Table 1, depressive symptom severity (BDI-II) showed selective and modest associations with maladaptive defense mechanisms, while no significant associations emerged with mature defenses. Specifically, higher depression scores were significantly and negatively correlated with dissociation (r = −0.211, *p* = 0.003) and denial (r = −0.160, *p* = 0.023), indicating that individuals with more severe depressive symptoms reported lower use of these two maladaptive defenses. The magnitude of these effects was small to moderate, with the strongest association observed for dissociation.

In contrast, projection was not significantly associated with depression (r = −0.108, *p* = 0.129), suggesting that depressive severity did not systematically relate to this defense mechanism. Likewise, no significant correlations were observed between depression and the mature defenses of humor (r = 0.096, *p* = 0.175), anticipation (r = 0.112, *p* = 0.115), or sublimation (r = 0.067, *p* = 0.342), indicating that depressive symptom severity was not meaningfully related to adaptive defensive functioning in this sample.

Overall, the pattern of results contradicts the expectation of a positive linear association between depression and maladaptive defenses and instead suggests a “defensive collapse” pattern, in which denial and dissociation decrease as depressive severity increases.

The hypothesis that an increased level of depressive symptoms is associated with a more frequent use of maladaptive defense mechanisms–was chosen based on clinical observations and conclusions from the specialized literature, which highlight the link between depression and the coping strategies adapted by the non-clinical population. The specialized literature reveals that depressed people tend to use dysfunctional mechanisms such as denial, projection or dissociation, as forms of protection against psychological suffering (Cramer, 2006; Vaillant, 1994). These mechanisms, although they may function for a certain period, contribute to the maintenance of emotional difficulties and may accentuate depressive symptoms in the absence of adaptive strategies learned over time. In contrast, mature defense mechanisms, such as sublimation, anticipation or humor, are increasingly rarely identifiable among people with severe depression, as they require a level of psychic reintegration and functional emotional resources learned throughout psychotherapy sessions (Bond, 2004; Gavin et al., 1993). The choice of this hypothesis is based on the intention to discover to what extent the severity of depression influences the psychological profile and how significant analogies can be identified to support psychotherapeutic interventions aimed at the development of more adaptive mechanisms. Thus, the hypothesis has both theoretical value and practical applicability in understanding and optimizing the treatment of major depressive disorder.

The results obtained in the correlation contradict the expectations that the level of depressive symptoms is associated with the type of defense mechanisms used, meaning that people with higher levels of depression may either resort more frequently to maladaptive mechanisms. Although it was exposed that people with high levels of depression will resort more frequently to dysfunctional defense mechanisms from various clinical experiences, the data indicate an unexpected relationship. The significant negative correlations between the BDI-II score and the use of dissociation (r = −0.211, *p* = 0.003), respectively, of denial (r = −0.160, *p* = 0.023), emphasize the fact that, as depressive symptoms become predominant in human behavior, these mechanisms are used less. A possible justifiable explanation would be the fact that, in the context of severe depression, psychic resources have reached the limit and there is no longer readaptation to go through situations, which reduces the individual’s capacity to be motivated.

Also, these defensive mechanisms could be explained by a direct exposure to psychic pain, without the possibility of avoidance or distortion strategies. Regarding projection, although it was assumed that although the frequent mechanism appeared during the depression period, no statistically significant correlation was identified (r = −0.108, *p* = 0.129), which indicates a possible interindividual differentiation in the coping strategies used. Thus, the supposition that the level of depressive symptoms is associated with the type of defense mechanisms used, meaning that people with higher levels of depression may either resort more frequently to maladaptive mechanisms is not supported by the results obtained, and the data suggest that the relationship between the evaluated variables is much more complex than we would have thought.

This identified phenomenon was deepened by the literature, which revealed that in severe stages of depression, the individual’s defensive system collapses, reducing the ability to mobilize intrinsically and extrinsically (Perry & Bond, 2012). According to Cramer, the symptomatic manifestations of major depression decrease the flow of defense mechanisms, because psychic resources are consumed, and the individual no longer manages to distort or avoid the surrounding reality (Cramer, 2006). Also, research by Sanchez et al. 2019 (as cited in Strauser et al., 2019, p. 99), has demonstrated that depression is often associated with a defensive-passive style, marked by direct exposure to suffering, without the intervention of active protective mechanisms.

Regarding dissociation, Becker-Nehring say that this phenomenon is much more pronounced in personality disorders or PTSD, and not necessarily in major depression, which may explain the low scores of this mechanism in the sample used in the research (Becker-Nehring et al., 2012). The lack of a significant correlation between depression and projection can be explained by the fact that depressed people tend to internalize conflicts rather than project them onto the outside world, a point of view supported by Fonagy and Luyten (Elliott & Prager, 2016), who complemented this perspective with the cognitive difficulties encountered and the tendencies to self-incrimination among people diagnosed with depression. Also, the individual variability of defensive responses, highlighted by Panfil (Panfil et al., 2020), indicates that not all individuals react through the same mechanisms, which supports the idea of a special relationship between the severity of depression and defensive style.

The assumption that anxious attachment style is associated with increased use of the defense mechanism of projection is based on the premise that anxious attachment style is interdependent with the use of projection as a defense mechanism, and the level of depression shapes this relationship. At the same time, it is expected that people with a predominant anxious attachment style will resort to projection to cope with interpersonal anxiety, and this effect will be influenced by the severity of depressive symptoms, so that depression could increase or decrease this effect. It is also analyzed how anxious attachment style interacts with depressive symptomatology and the use of projection, in the sense that people with high anxious attachment could resort more frequently in contexts of heightened emotional distress. Thus, a tendency for interaction between anxious attachment style and the level of depression is expected, even if this could be of reduced intensity.

Scientific research emphasizes that adults with an anxious attachment style experience a constant fear of abandonment, hypervigilance to rejection, and an excessive need for approval and emotional and cognitive reassurance (Mikulincer & Shaver, 2007).

Furthermore, depression, through its disruptive effect on emotional self-regulation and self-perception, can influence the use of defense mechanisms in a complex way. Some studies have shown that the severity of depressive symptoms can intensify the use of projection in individuals with anxious attachment, because cognitive distortions associated with depression (e.g., self-devaluation tendencies or excessive personalization) contribute to the exacerbation of intrapsychic conflicts (Fonagy & Luyten, 2016). Other research suggests that in severe stages of depression, some defense mechanisms may become inactive or ineffective, which could modulate the effect of anxious attachment on projection (Perry & Bond, 2012).

In this sense, Table 2 addresses the results of the analysis that had as its principle the measurement of anxious attachment style, the level of depression and the interaction between the two can predict the use of projection. This structured analysis allows the identification of the individual and combined influences of attachment and depression on the tendency to project psychic contents onto others.

The overall model predicting projection from anxious attachment, depression, and their interaction was significant, R^2^ = 0.05, F(3,196) = 3.09, *p* = 0.028. Anxious attachment positively predicted projection (B = 4.654, *p* = 0.007), whereas the Anxious × Depression interaction was negative and significant (B = −0.099, *p* = 0.005), indicating that the positive association between anxious attachment and projection weakened at higher levels of depressive symptoms.

As a result of the regression analysis, the results show that anxious attachment is significantly associated with projection (B = 4.654, *p* = 0.040), which partially supports the hypothesis, in the sense that people with anxious attachment style resort to projection at different times, greatly depending on the diversity of personalities, but also on the way in which they interacted with the family environment and the relationships they have founded. This result is in line with the literature, which indicates that anxious individuals in relationships may use projection to manage emotional uncertainty and fear of rejection (Fraley et al., 2021). Contrary to expectations, the depression variable did not model the use of projection (B = 0.192, *p* = 0.300), which indicates that, in this model, depressive symptoms do not explain variations in the use of this defense mechanism.

Also, the interaction between anxious attachment and depression was not statistically significant (B = −0.099, *p* = 0.088), although it approaches the threshold of significance. Thus, we cannot state that depression definitely moderates the relationship between attachment and projection; but we can highlight the moderation trend, which could suggest a reduction in the influence of anxious attachment on projection. Therefore, the component related to anxious attachment is supported by the data, while the effect of depression and the interaction between the variables do not reach statistical significance. However, the results offer future perspectives for analysis and reflection.

According to the studies, researchers found that individuals with anxious attachment show increased activation of projection and negative thoughts in situations perceived as threatening, and this activation is often automatic (Fonagy & Luyten, 2016). Similarly, it was identified that anxious people tend to frequently use projection as an unconscious strategy to reduce emotional-cognitive dissonance in attachment relationships, especially if the experiences lived with the family of origin were based on rejection and abandonment in critical situations (Tanzilli et al., 2021). Also, Tanzilli observed an affiliation of the level of anxiety depending on the attachment person may contribute to a distorted perception in relationships with others, as individuals tend to attribute their own negative feelings to those around them to reduce internal tension (Prunas et al., 2019; Tanzilli et al., 2021).

If we assume that the formation of clusters of participants in which avoidant attachment and high depression are associated with frequent use of denial, one of the clusters is expected to highlight a specific psychological profile, in which participants with high depression scores and a pronounced avoidant attachment style show an increased frequency of using denial as a defense mechanism. This profile would suggest a specific defensive mechanism, in which the individual, faced with significant emotional discomfort, resorts to denial to avoid affective reality and thus protect his psychological integrity.

Table 3 presents the final centers of the two clusters identified in the clustering analysis, according to depression scores, avoidant attachment style and use of the denial defense mechanism. This table reflects the mean values for each of the variables analyzed in the two groups, highlighting the significant differences between them.

The aim of the analysis was to explore whether there are distinct clusters of participants who combine high depression scores, an avoidant attachment style and a frequent use of the defense mechanism of denial. It is anticipated that one of these clusters will show a significant association between high levels of depression, a pronounced avoidant attachment style and an increased frequency of use of denial, which would suggest a defensive profile. By analyzing these clusters, we then identified groups of participants who in their everyday behavior display this complex combination of traits.

To provide a comprehensive understanding of significant behavioral patterns, a clustering analysis (k-means method) was applied, having as variables of interest depression scores (BDI-II), denial defense mechanism and avoidant attachment style. Cluster 1 is characterized by a moderate level of depression, a high score of the denial defense mechanism and a slightly accentuated avoidant attachment style. In contrast, cluster 2 brings together participants with a severe level of depression, a lower denial score and a slightly diminished avoidant attachment compared to the first cluster. These results suggest the existence of two distinct psychological profiles, differentiated mainly by the severity of depression and the predominant defensive strategy used.

Table 4 highlights the distribution of cases across the two identified clusters. The purpose of this table is to highlight the number of participants in each cluster, allowing an objective assessment of the distribution of the data collected and the representativeness of the groups involved in the research. Thus, we can assess whether one of the groups supports the hypothesis that avoidant attachment combined with depression would be correlated with frequent use of denial.

To understand the distribution of participants according to the identified psychological profiles, the number of cases in each cluster was analyzed. Table 4 presents the results of the k-means cluster analysis, which identified two distinct participant profiles based on depressive severity, denial, and avoidant attachment. The two clusters differed significantly on all three variables, with moderate to large effect sizes. It highlights that, out of a total of 200 participants included in the analysis, 134 were classified in group 1, and 66 in group 2. Cluster 1 (*n* = 134) was characterized by moderate depressive symptom severity (M ≈ 22.90, SD ≈ 15.26), higher levels of denial (M ≈ 47.40, SD ≈ 15.27), and higher avoidant attachment (M ≈ 3.75, SD ≈ 0.86). Cluster 2 (*n* = 66) showed significantly more severe depressive symptoms (M ≈ 38.12, SD ≈ 19.41), accompanied by significantly lower denial (M ≈ 39.59, SD ≈ 18.09) and lower avoidant attachment (M ≈ 2.76, SD ≈ 1.44). The presence of these two clusters suggests not only a quantitative, but also a qualitative differentiation in the way individuals cope with emotional distress, offering useful perspectives for differentiated psychological interventions.

Table 5 presents the analysis of variance (ANOVA) for BDI-II depression scores, denial and avoidant attachment between the two identified clusters. Next, we will analyze the results obtained for each of these variables, in order to assess significant differences between the groups and to better understand how the studied variables relate in the context of the identified cluster.

The results indicate a significant difference between the two groups included in the proposed analysis, confirming that the severity of depression significantly differentiates the two groups. Cluster 2, with a score of 50, indicates severe depression, compared to Cluster 1, which has a score of 17. These differences support the idea that the severity of depression influences responses regarding denial. The ANOVA test marked a significant difference (F = 10.205, *p* = 0.002), indicating that denial is used more frequently in Cluster 1 (score 47) than in Cluster 2 (score 40). This suggests that people with less severe depression resort to denial more frequently, while those with severe depression choose other coping strategies (Bohlmeijer et al., 2011). Also, for avoidant attachment, ANOVA showed a significant difference, which suggests a tendency of people with moderate depression to adopt this attachment style (Mikulincer & Shaver, 2007).

This finding is supported by the fact that individuals with moderate depression more frequently resort to denial to reduce their emotional discomfort, while in severe forms of depression, defensive resources are diminished, and the individual can no longer support mechanisms of avoidance or distortion of reality, confronting more directly with psychological suffering. This result is in agreement with the specialized literature that shows that defense mechanisms such as denial can function effectively only in an early phase of depressive symptomatology, being less present in severe cases (White et al., 2013).

In addition to the significant differences identified between levels of depression and the use of denial, recent literature provides additional arguments in support of the idea that the severity of depression influences the choice of defense mechanisms. This is explained by the depletion of cognitive and emotional resources that allow the maintenance of functional defense mechanisms in early stages of depression. Thus, denial seems to be an effective mechanism in mild to moderate stages of depression, but in severe forms, the individual no longer has the capacity to support it, being overwhelmed by what is happening and reflecting a passive acceptance of pain rather than an avoidance of it. These results complement the data present in our analysis, suggesting that defense mechanisms are sensitive to the severity of affective disorders and can function as indicators in establishing the level of psychological impairment (Di Giuseppe et al., 2024).

If we assume that women will score higher in the use of dysfunctional defense mechanisms compared to men and that depression will significantly increase this use in women. Depression is expected to increase the use of dysfunctional defense mechanisms, especially in women, which would address a potential link between psychological vulnerability and gender in the context of affective disorders. Table 6 presents the summary of the regression analysis, highlighting the relationship between the independent variables (Gender_Depression_Interaction and Gender_Dummy) and the dependent variable, along with the adjustment parameters and the standard error of the estimate (it examines the effects of gender, depressive severity, and their interaction on the use of dysfunctional defense mechanisms.

The overall regression model was statistically significant, explaining a small but meaningful proportion of variance in dysfunctional defenses (R^2^ ≈ 0.047, *p* < 0.01). The main effect of gender was significant and positive (B ≈ 15.5, *p* < 0.05), indicating that one gender group reported significantly higher levels of dysfunctional defensive mechanisms compared to the reference group. In contrast, depressive severity alone did not emerge as a strong direct predictor once the interaction was included in the model. Crucially, the gender × depression interaction was negative and statistically significant (B ≈ −0.58, *p* < 0.01), demonstrating that the relationship between depressive symptoms and dysfunctional defenses differs by gender. Specifically, as depressive severity increased, the level of dysfunctional defenses decreased more steeply in the reference gender group, whereas in the comparison group this decline was substantially attenuated. Although the coefficient of determination indicates that the model predictors explain only a small part of the variability of dysfunctional defense mechanisms, the analysis was continued by applying the ANOVA test to verify whether what was assumed is statistically significant.

Also, Table 7 presents the analysis of variance (ANOVA) for the regression model of dysfunctional defense mechanisms. We will analyze the significance of the results and their implications in the context of the research.

The results of the ANOVA analysis indicate that it is statistically significant, suggesting that variables such as gender, depression, and their interaction have a real impact on the use of dysfunctional defense mechanisms, although the effect is relatively small. These results indicate a significant relationship between gender, depression, and the use of defense mechanisms, even if the explanation of the variance is limited. The interaction of the variables suggests that the effects of depression on the formation of defense mechanisms are modulated by the gender of the participants. Recent studies support this hypothesis, showing that women with depression tend to use passive-internalizing defense mechanisms, such as rumination and avoidance, while men may resort to more aggressive and externalizing mechanisms (Piccinelli & Wilkinson, 2000; Walther, 2025). This may explain why depression influences the use of dysfunctional defense mechanisms more in women than in men.

Table 8 shows that the interaction between gender and depressive severity significantly predicted dysfunctional defense mechanisms, indicating that the association between depression and dysfunctional defenses differs by gender. Specifically, as depressive symptoms increased, dysfunctional defenses decreased more strongly in one gender group, confirming a moderating effect of gender rather than a simple main effect.

To analyze the impact of gender and the interaction between gender and depression on the use of dysfunctional defense mechanisms, a linear regression model was applied. The aim was to determine whether these variables have a significant effect on the use of dysfunctional defense mechanisms, given that the literature suggests that gender differences and depression can influence coping strategies and the use of defense mechanisms.

The results of the analysis confirm that both gender and the interaction between gender and depression significantly influence the use of dysfunctional defense mechanisms. Men have higher scores in the use of these mechanisms (B = 15.485, *p* = 0.036), which is consistent with previous studies suggesting that men more frequently adopt externalizing and aggressive defense strategies (Vaillant, 1998). The interaction between gender and depression also showed that depression reduced the use of dysfunctional defense mechanisms in women (B = −0.579, *p* = 0.002), supporting research suggesting that women with depression use internalizing defense mechanisms, such as rumination and dissociation (Calati et al., 2008). These results highlight that the effect of depression on defense mechanisms is modulated by gender, which is consistent with Silverman observations that biological and cultural factors interact in how women and men cope with emotional distress (Silverman & Aafjes-van Doorn, 2023).

The distribution of the study variables was examined using the Shapiro–Wilk and Kolmogorov–Smirnov tests. The results indicated significant deviations from normality for most of the variables included in the correlation and regression analyses (all *p* < 0.05), including depressive symptom severity, attachment dimensions, defense mechanisms, and interaction terms. However, given the large sample size (N = 200), parametric statistical procedures were retained, as correlation and regression analyses are considered robust to violations of normality under such conditions. This decision is consistent with recommendations derived from the Central Limit Theorem. The normality of regression residuals was examined using the Shapiro–Wilk and Kolmogorov–Smirnov tests. For the regression predicting projection from anxious attachment, depressive symptoms, and their interaction, the Shapiro–Wilk test indicated a modest deviation from normality (W = 0.97, *p* < 0.001), whereas the Kolmogorov–Smirnov test on standardized residuals was non-significant (D = 0.07, *p* = 0.28). Similarly, for the regression predicting dysfunctional defense mechanisms from gender and the gender × depression interaction, the Shapiro–Wilk test was significant (W = 0.98, *p* = 0.013), while the Kolmogorov–Smirnov test was non-significant (D = 0.07, *p* = 0.27). Given the large sample size (N = 200) and the robustness of linear regression to moderate deviations from normality, all regression analyses were retained.

Regression assumptions were formally tested prior to interpretation of the models. Multicollinearity was evaluated using the variance inflation factor (VIF), which indicated no problematic collinearity among predictors in either regression model (all VIF values < 3.2). Homoscedasticity of residuals was examined using the Breusch–Pagan test and was supported for both models (all *p* > 0.26). Independence of errors was verified using the Durbin–Watson statistic, with values of 1.52 for the projection model and 1.46 for the dysfunctional defense model, indicating acceptable error independence. Although the Shapiro–Wilk test indicated mild deviations from normality of residuals, regression analyses were retained due to the large sample size and the robustness of linear models to moderate violations of normality.

## 4. Discussion

The study examined the relationships between depression severity, attachment styles, and the use of defense mechanisms in individuals with different levels of depression. The goal was to better understand how these variables are related and to make important contributions to the field of treatment and intervention for depression. The results revealed both confirmations of hypotheses from the existing literature and new findings that contradict initial expectations (Cramer, 2006; Mikulincer & Shaver, 2007).

Depression and maladaptive defense mechanisms: We hypothesized that severe depression would be associated with a frequent use of dysfunctional defense mechanisms, such as denial, projection, and dissociation. However, the correlation analysis showed unexpected results. Instead of a positive correlation, we found significant negative correlations between depression severity and the use of dysfunctional defense mechanisms. Dissociation was identified a significant negative correlation (r = −0.211, *p* = 0.003), which helped us to deduce that individuals with severe depression use this defense mechanism less compared to those with milder depression (Vaillant, 1998). Denial: also, the correlation identified in the analysis (r = −0.160, *p* = 0.023) suggests that individuals with severe depression are less likely to deny emotional reality, which may reflect a greater awareness of their own emotions and symptoms (Freud & Institute of Psycho-analysis (Great Britain), 1993; Mikulincer & Shaver, 2007).

These results contradict the hypothesis that severe depression would be correlated with a more frequent use of maladaptive defense mechanisms. Instead, it is suggested that people with severe depression may show greater awareness of their emotional reality, which may make them less likely to resort to defense mechanisms that deny or distort reality. It is possible that these people have a more direct approach to their symptoms, preferring to confront or accept them rather than avoid them through dysfunctional mechanisms (Cramer, 2006).

Regarding the analysis of adaptive defense mechanisms, such as humor, anticipation, and sublimation, no significant correlations were found between the severity of depression and their use. Although the hypothesis from which we started brought to the fore the symptomatologic and behavioral manifestation of people with depression, from which we intuited that they must resort to mature defense mechanisms, such as sublimation, the results suggest that these mechanisms are not frequently used in the case of severe depression. This may indicate significant emotional difficulties, which could contribute to the maintenance of depressive symptoms. Individuals with severe depression are likely to lack access to or the ability to apply these more adaptive mechanisms, due to the severity of their symptoms (Gross, 2002).

Attachment styles and defense mechanisms: ANOVA analysis was used to examine differences between clusters of participants in order to better understand how the use of defense mechanisms differs according to the severity of depression and attachment styles. The results of the study highlighted notable differences between the groups affected by severe depression and those in which depression is easier to bear, in terms of the use of denial and the avoidant attachment style. Fascinatingly, people who experience a severe form of depression seem to approach things differently: they use denial less, which could lead us to think that they have a deeper awareness of their own emotions. In contrast, individuals struggling with milder depression tend to apply denial more frequently. This could suggest that they rely on insecure attachment styles, such as avoidant. This dynamic suggests that attachment styles that are not stable are associated with an increased use of defense mechanisms designed to diminish or avoid confronting emotional reality (Mikulincer & Shaver, 2007). These findings align perfectly with previous studies, which show that people with insecure attachment styles, especially avoidant ones, often opt for dysfunctional defense mechanisms, such as denial.

Gender differences and their influence on defense mechanisms: One of the significant findings of this study is related to gender differences in the use of defense mechanisms, especially in the context of depression. The results suggest that there are significant differences between men and women in the types of defense mechanisms used. Men tend to more frequently adopt defense mechanisms that are oriented towards external behavior, such as dissociation and externalizing behaviors of emotions. These mechanisms are used to mask internal vulnerabilities and to prevent confronting feelings of helplessness or sadness. Men with severe depression are more likely to externalize emotions through aggression or impulsive behaviors (Corrigan et al., 2014). Women, on the other hand, tend to use defense mechanisms that are directed towards internal behaviors, such as rumination and dissociation. These mechanisms are associated with a more introspective approach, but they can contribute to the intensification of depressive symptoms, as women may become immersed in constant reflection on their own emotional state, which can amplify negative thought cycles (Mikulincer & Shaver, 2007)

These gender differences highlight cultural and social influences that may determine how men and women are taught to manage their emotions and cope with emotional distress. For example, in many cultures, men are expected to be less external and to engage in aggressive behaviors, while women are often encouraged to internalize and express emotions in a more passive or ruminative manner (Vergara-Lopez et al., 2024).

The results of this study suggest that depression severity, attachment styles, and defense mechanisms are closely interconnected, and understanding these relationships may have significant implications for the development of psychotherapeutic intervention strategies (Békés et al., 2021). The findings suggest that individuals with severe depression may benefit from interventions that support the development of more effective and adaptive coping mechanisms (Everaert & Joormann, 2019). Furthermore, gender differences in the use of defense mechanisms highlight the importance of personalizing therapeutic approaches according to gender and the cultural and social factors that influence how depression is emotionally managed (Hyde et al., 2008; Kwon et al., 2013).

The study highlights the importance of an accurate diagnosis of the defense mechanisms used by people with depression, in the context of their attachment style. Depending on the results obtained, psychotherapeutic interventions should be personalized to meet the specific needs of each individual:

- for people with secure attachment: these people may benefit from interventions that promote the consolidation of their emotional resources and the development of adaptive defense mechanisms. Psychotherapists should focus on strengthening emotional self-regulation and stimulating positive behavior (Kobak & Sceery, 1988; Mikulincer & Shaver, 2007);
- for people with avoidant attachment: These people may have difficulty coping with emotions and intimate relationships. Interventions should help reduce the use of maladaptive defense mechanisms, such as denial or dissociation, and support the construction of more stable and healthier interpersonal relationships (Bosmans & Borelli, 2022; Mikulincer & Shaver, 2007);
- for people with anxious attachment: In this case, psychotherapy may include techniques to help reduce anxiety and promote a more balanced attachment. It is also important to address tendencies to use intermediate defense mechanisms, such as rationalization or repression, and to encourage healthy emotional expression (Cassidy & Shaver, 2018; Main & Solomon, 1990).

These results suggest that psychotherapists should use instruments to assess attachment style and defense mechanisms, personalizing interventions according to the individual typology of the client (Sroufe, 2005).

The research findings highlight the need for a nuanced psychotherapeutic approach that takes into account the complex psychodynamic and relational profile of each client, especially regarding the interaction between attachment style, depression level and defense strategies. This approach supports a truly personalized intervention, with significant benefits on the effectiveness of the therapeutic process.

1. Comprehensive initial assessment: the initial psychological assessment must integrate deep dimensions of psychological functioning, not just manifest symptomatology. Identification of attachment style through tools such as Experiences in Close Relationships (ECR-R) (Fraley et al., 2021; Fraley & Shaver, 2000) and defense mechanisms through the Defense Style Questionnaire (DSQ-40) (Gavin et al., 1993; Kramer et al., 2009) allows for the outline of a complete picture of the client’s vulnerabilities and resources. According to a recent meta-analysis (Dagan et al., 2018), insecure attachment style is directly associated with an increased risk of major depressive disorder, and defense mechanisms play a mediated role in this relationship;
2. Adapting the therapeutic style: the therapeutic style must be adapted to meet the specific emotional and relational needs of each client:
  - anxious attachment: It is characterized by hypersensitivity to rejection, fear of abandonment and the tendency to merge with the attachment figure. In this case, a predictable therapeutic framework, with a relationship marked by validation and stability, is essential. Techniques from Dialectical-Behavioral Therapy (Linehan, 2025) or Emotion-Focused Therapy (Greenberg, 2017) can be useful for regulating intense emotions.
  - avoidant attachment: These people tend to reject deep emotional contact and deny affective needs. The therapeutic approach should be empathetic, indirect, but constant, facilitating gradual access to emotions. Mentalization-Based Therapy (Fonagy & Luyten, 2016) is recommended because it allows the exploration of affect in a tolerable way for the client.

For clients with avoidant attachment and tendencies towards dissociation or isolation, a gradual approach is necessary, focused on building trust and accessing emotions at a tolerable pace, given that attachment structures profoundly influence the way the individual relates to suffering and the therapeutic relationship (K. Levy et al., 2015; K. N. Levy et al., 2019). Recent studies confirm that adapting the therapeutic style to the attachment profile leads to a stronger therapeutic alliance and a faster reduction in depressive symptomatology (Zimmermann, 2022).

**Figure 1:**
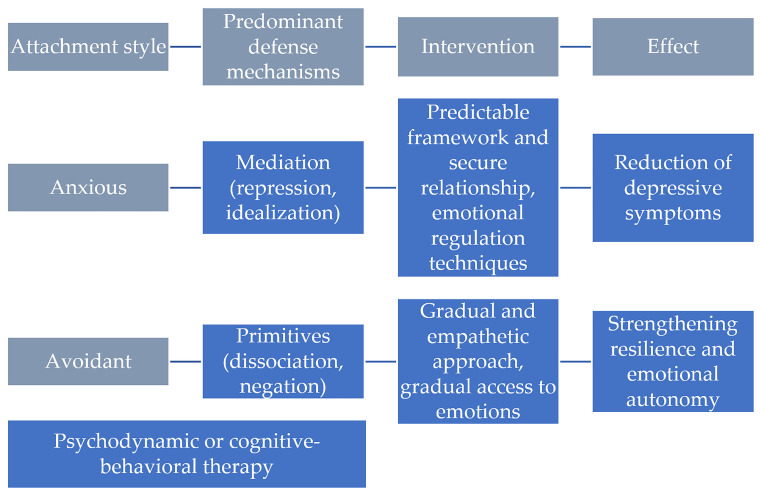
The interaction between attachment style, defense mechanisms and clinical depression in psychotherapy.

To highlight the complexity of the process of personalized psychotherapeutic intervention, depending on the attachment style and predominant defense mechanisms, Figure 1 summarizes the key stages of clinical adaptation. This visual approach is inspired by the model proposed by Luyten & Fonagy, which emphasizes the interdependence between attachment, personality, and psychopathology, and is adapted according to the results of the present research (Luyten & Fonagy, 2022).

Figure 1 presents two different therapeutic pathways, constructed according to attachment style, predominant defense mechanisms, and individually tailored interventions:

- People with an anxious attachment style, characterized by a fear of abandonment and a strong need for closeness, often use intermediate defense mechanisms, such as repression and idealization. In the case of these clients, research indicates that a safe and predictable therapeutic relationship is essential for the healing process. Interventions focused on emotional regulation, such as those within Emotion-Focused Therapy (Greenberg, 2017) or Schema Therapy (Young, 2006), can facilitate the processing of intense emotions and the restructuring of representations of self and others. Also, validating emotional experiences and using “guided experiential” techniques can reduce the level of relational anxiety and the intensity of depressive symptoms (Antuña-Camblor et al., 2024);
- People with an avoidant attachment style, who tend to suppress emotions and avoid intimate connections out of fear of vulnerability, frequently rely on primitive defense mechanisms, such as dissociation or denial. In their case, a gradual and empathetic approach is essential, with an emphasis on accessing emotions at a tolerable pace, to avoid retraumatization or breaking the therapeutic alliance. Therapies such as Mentalization-Based Therapy (Fonagy & Luyten, 2016) or integrated attachment-focused interventions, proposed in the recent literature (Bateman & Fonagy, 2019), have proven effective in increasing the capacity to reflect on one’s own and others’ mental states.

3. Interventions targeting defense mechanisms: defense mechanisms can significantly influence the course of therapy. A defensive profile dominated by primitive defenses (e.g., dissociation, projection) requires a slow, psychodynamic approach, centered on awareness of unconscious internal conflicts:
  - insight-focused psychodynamic therapy (Ciocca et al., 2020) can help transform maladaptive mechanisms into more mature forms (sublimation, anticipation);
  - cognitive-behavioral therapy (J. S. Beck & Beck, 2021) focuses on restructuring dysfunctional thoughts and can help clients develop alternative coping strategies, reducing reliance on rigid defense mechanisms.

A longitudinal clinical study (Alford & Beck, 1997) showed that reducing immature defense mechanisms in therapy is associated with a significant decrease in depression scores 6 months post-intervention.

4. Interventions based on mentalization and emotional validation: in the case of people with insecure attachment styles, working on mentalization (the ability to understand one’s own and others’ mental states) is essential for developing a stable identity and a healthy relationship with the self:
  - Mentalization-Based Therapy (Fonagy & Luyten, 2016) is effective for clients who oscillate between emotional hyperactivation (anxious) and affective inhibition (avoidant).
  - Emotion-Focused Therapy (Greenberg, 2017) promotes contact with basic emotions and the transformation of maladaptive emotions through corrective emotional experience.
5. Long-term implications: a personalized therapeutic approach, which integrates the attachment profile and defensive patterns, contributes not only to the reduction in depressive symptomatology, but also to the development of psychological resilience and emotional autonomy in the long term. In addition, the strengthening of reflective functions and the capacity for self-regulation provides a solid basis for the prevention of relapse. According to Dimaggio, “interventions that simultaneously address attachment, affect, and defense mechanisms are those that have the greatest impact on personality restructuring and the reduction in persistent depression” (Dimaggio et al., 2013).

## 5. Conclusions

The present research explored the associations between depressive symptoms, defense mechanisms, and attachment styles, with attention to gender and severity effects. The findings reveal several patterns that both align with and diverge from results reported in the past decade of research articles.

Recent literature consistently emphasizes that depression is associated with an increased reliance on immature defense mechanisms and a diminished use of mature ones. Large-scale and clinical studies have repeatedly demonstrated that individuals with higher depressive symptomatology tend to employ defenses such as projection, denial, and dissociation more frequently, while showing lower reliance on adaptive strategies such as sublimation or humor (Di Giuseppe et al., 2024; Fiorentino et al., 2024). These findings converge with network analyses indicating that immature defenses occupy a central position within depressive and anxious symptom constellations.

Contrary to this well-established pattern, the current results showed negative correlations between depression and both denial and dissociation, suggesting that individuals with more severe depressive symptoms displayed less reliance on these immature defenses. This divergence may point to a phenomenon of defensive collapse or exhaustion at higher levels of affective disturbance, in which the capacity to maintain defensive distortion diminishes as depressive severity increases. Although this possibility has been discussed conceptually in psychoanalytic and psychodynamic literature, few empirical studies in the past decade have provided direct support for it. The present data thus offer a novel quantitative indication that defensive functioning may decline rather than intensify at severe depressive levels.

Consistent with attachment theory and prior meta-analyses, anxious attachment was positively associated with depressive symptoms (Dagan et al., 2018; Zhang et al., 2022) and, more specifically, predicted increased use of projection. The linkage between anxious attachment and projection aligns with theoretical taxonomies that classify projection among the “depressive” or affectively charged defenses (Di Giuseppe et al., 2024), yet direct empirical demonstrations of this relationship remain rare in the recent literature. The absence of a moderation effect by depression further refines the understanding of this pathway, indicating that anxious attachment exerts its influence on defensive style relatively independently of depressive severity. This null moderation result complements existing models by defining a boundary condition rarely tested in prior work.

The exploratory cluster analysis identified two distinct profiles: one group with moderate depression characterized by higher denial and slightly higher avoidant attachment, and another with severe depression showing lower denial and lower avoidant attachment. These profiles reinforce the inverse relationship between depressive severity and denial and suggest that avoidance may operate differently across levels of depression. Comparable multivariate clustering approaches linking depression, attachment, and specific defenses are scarcely reported in the 2015–2025 literature (see, e.g., Di Giuseppe et al., 2024). Thus, this pattern represents an empirically new contribution that extends beyond the traditional bivariate frameworks predominant in the field.

Gender differences in defense mechanisms have been intermittently observed in recent research, with some studies reporting that men exhibit higher levels of dysfunctional or immature defenses compared to women (Bullitt & Farber, 2002). The current results replicate this tendency, but also reveal a significant gender × depression interaction, whereby increasing depression was associated with a reduction in dysfunctional defenses among women. This direction of interaction is uncommon in the existing literature and may indicate gender-specific patterns of defensive modulation under depressive stress. Although the overall effect was small, it introduces an exploratory nuance warranting replication.

Collectively, the findings are partly consonant with, yet partly divergent from, the broader empirical landscape. The correlations between anxious attachment and projection align with established theoretical expectations, whereas the observed decrease in certain immature defenses (denial and dissociation) as depression worsens challenges prevailing models that assume linear increases in defensive immaturity. Together with the identified cluster structure and the gender-specific moderation, these results suggest that the relationship between depression and defensive functioning is non-linear and may vary across severity levels and individual characteristics.

In summary, when situated against the past decade of published literature, this study contributes three potential advances. First, it provides empirical evidence for a reduction in certain immature defenses at high depressive severity, a finding that departs from the dominant positive associations reported in prior research (Di Giuseppe et al., 2024; Fiorentino et al., 2024). Second, it offers specific empirical support for the link between anxious attachment and projection independent of depression, a relationship theoretically posited but rarely tested. Third, it identifies a gender-related moderation of defensive functioning that has not been systematically documented in the recent literature. These contributions underscore the importance of considering depression severity, attachment dimensions, and gender when modeling defensive patterns, and highlight the need for longitudinal and clinical replication to determine whether the observed “defensive collapse” represents a stable phenomenon or a sampling artifact.

## Figures and Tables

**Table 1:** Correlations between depression scores and measured defense mechanisms.

		Depression Score BDI II	Humor Score	Anticipation Score	Sublimation Score	Projection Score	Denial Score	Dissociation Score
Depression score BDI_II	Pearson Correlation	1	0.096	0.112	0.067	−0.108	−0.160 *	−0.211 **
	Sig. (2-tailed)		0.175	0.115	0.342	0.129	0.023	0.003
	N	200	200	200	200	200	200	200
Humor score	Pearson Correlation	0.096	1	0.087	−0.140 *	−0.011	0.101	−0.007
	Sig. (2-tailed)	0.175		0.219	0.047	0.873	0.154	0.920
	N	200	200	200	200	200	200	200
Anticipation score	Pearson Correlation	0.112	0.087	1	−0.107	−0.084	−0.100	−0.102
	Sig. (2-tailed)	0.115	0.219		0.132	0.238	0.157	0.152
	N	200	200	200	200	200	200	200
Sublimation score	Pearson Correlation	0.067	−0.140 *	−0.107	1	−0.198 **	−0.121	0.009
	Sig. (2-tailed)	0.342	0.047	0.132		0.005	0.089	0.902
	N	200	200	200	200	200	200	200
Projection score	Pearson Correlation	−0.108	−0.011	−0.084	−0.198 **	1	0.176 *	0.253 **
	Sig. (2-tailed)	0.129	0.873	0.238	0.005		0.013	0.000
	N	200	200	200	200	200	200	200
Denial score	Pearson Correlation	−0.160 *	0.101	−0.100	−0.121	0.176 *	1	0.126
	Sig. (2-tailed)	0.023	0.154	0.157	0.089	0.013		0.075
	N	200	200	200	200	200	200	200
Dissociation score	Pearson Correlation	−0.211 **	−0.007	−0.102	0.009	0.253 **	0.126	1
	Sig. (2-tailed)	0.003	0.920	0.152	0.902	0.000	0.075	
	N	200	200	200	200	200	200	200

* Correlation is significant at the 0.05 level (2-tailed); ** Correlation is significant at the 0.01 level (2-tailed).

**Table 2:** Analysis of regression coefficients for projection score according to anxious attachment style and depression.

Coefficients ^a^										
Model		Unstandardized Coefficients		Standardized Coefficients	t	Sig.	95.0% Confidence Interval for B		Collinearity Statistics	
		B	Std. Error	Beta			Lower Bound	Upper Bound	Tolerance	VIF
1	(Constant)	35.677	7.028		5.076	0.000	21.815	49.538		
	Anxious attachment score	4.654	2.249	0.284	2.070	0.040	0.219	9.089	0.262	3.812
	BDI II depression score	0.192	0.184	0.208	1.040	0.300	−0.172	0.555	0.124	8.089
	Anxious Style-Depression Interaction	−0.099	0.057	−0.408	−1.715	0.088	−0.212	0.015	0.087	11.465

^a^ Dependent Variable: Projection score.

**Table 3:** The final centers of the investigated groups.

	Cluster	
	1	2
Depression score BDI_II	17	50
Denial score	47	40
Avoidant attachment score	4	3

**Table 4:** Distribution of cases by clusters.

		Number of Cases in Each Cluster
Cluster	1	134.000
	2	66.000
Valid		200.000
Missing		0.000

**Table 5:** Analysis of variance (ANOVA).

		Sum of Squares	df	Mean Square	F	Sig.
Depression score BDI_II	Between Groups	10,251.152	1	10,251.152	36.609	0.000
	Within Groups	55,443.568	198	280.018		
	Total	65,694.720	199			
Denial score	Between Groups	2693.528	1	2693.528	10.205	0.002
	Within Groups	52,261.992	198	263.949		
	Total	54,955.520	199			
Avoidant attachment score	Between Groups	43.226	1	43.226	36.655	0.000
	Within Groups	233.494	198	1.179		
	Total	276.720	199			

**Table 6:** Summary of the regression model for the investigated predictors.

Model	R	R Square	Adjusted R Square	Std. Error of the Estimate
1	0.217 ^a^	0.047	0.037	33.444

^a^ Predictors: (Constant), Gender_Depression_Interaction, Gender_Dummy.

**Table 7:** Analysis of variance (ANOVA) for the regression model of dysfunctional defense mechanisms.

			ANOVA ^a^			
Model		Sum of Squares	df	Mean Square	F	Sig.
1	Regression	10,837.576	2	5418.788	4.845	0.009 ^b^
	Residual	220,347.179	197	1118.514		
	Total	231,184.755	199			

^a^ Dependent Variable: Dysfunctional_Defense_Mechanisms. ^b^ Predictors: (Constant), Gender_Depression_Interaction, Gender_Dummy.

**Table 8:** Regression coefficients for dysfunctional defense mechanisms.

Coefficients ^a^						
Model		Unstandardized Coefficients		Standardized Coefficients	t	Sig.
		B	Std. Error	Beta		
1	(Constant)	137.864	3.189		43.234	0.000
	Gender_Dummy	15.485	7.353	0.227	2.106	0.036
	Gender_Depression_Interaction	−0.579	0.188	−0.332	−3.089	0.002

^a^ Dependent Variable: Dysfunctional_Defense_Mechanisms.

## Data Availability

The raw data supporting the conclusions of this article will be made available by the authors on request.
